# Assessment of Healthcare Provider Workload in Neonatal Resuscitation

**DOI:** 10.3389/fped.2020.598475

**Published:** 2020-12-22

**Authors:** Emily C. Zehnder, Brenda H. Y. Law, Georg M. Schmölzer

**Affiliations:** ^1^Centre for the Studies of Asphyxia and Resuscitation, Royal Alexandra Hospital, Edmonton, AB, Canada; ^2^Department of Pediatrics, University of Alberta, Edmonton, AB, Canada

**Keywords:** infant, workload, neonatal resuscitation, demands, cognitive load

## Abstract

**Objective:** Human errors or protocol deviations during neonatal resuscitation are common. Excess workload has been proposed as a contributor to human error during medical tasks. We aim to characterize healthcare providers' perceived workload during neonatal resuscitation.

**Design:** Perceived workload was measured using a multi-dimensional retrospective National Aeronautics and Space Administration Task Load Index (NASA TLX) survey. The NASA TLX collects data on mental, physical, and temporal demand, performance, effort, and frustration. Each section is rated independently by participants on a scale of 0–20 (0 being lowest and 20 being highest). The Raw-TLX score is a composite score of all dimensions and presented on a scale of 0–100. Healthcare providers complete a paper and pencil survey after attending delivery room resuscitations within 3 months.

**Setting:** Level three neonatal intensive care unit at the Royal Alexandra Hospital, Edmonton, AB, Canada.

**Participants:** All neonatal healthcare providers who attended deliveries.

**Exposure:** Participation in the delivery room care of newborns.

**Measurement:** Raw TLX scores as a measure of overall workload and scores for each dimension of workload.

**Main Results:** During the study period, ~880 neonatal resuscitation events occurred, and a total of 204 surveys were completed. Healthcare providers completed one survey for 179 deliveries, two surveys for 20 deliveries, and three surveys for 5 deliveries. The mean (standard deviation) gestational age was 35 (5) weeks, and the median (interquartile range) birth weight was 2,690 (1,830–3,440) g. Interventions at delivery were (i) stimulation 149 (73%), suction 130 (64%), continuous positive airway pressure 120 (59%), positive pressure ventilation 105 (52%), intubation 33 (16%), chest compression 10 (5%), and epinephrine 4 (2%). The overall median (interquartile range) Raw-TLX was 34 (18–49). The scores varied by dimension with mental demand 10 (5–14), physical demand 4 (1–6), temporal demand 8 (3–14), performance 4 (2–6), effort 8 (4–13), and frustration 4 (1–10). Raw-TLX scores were higher when healthcare providers performed any intervention compared to no intervention [35 (22–49) vs. 8 (6–18), *p* = 0.0011]; intubation and no intubation was [55 (46–62) vs. 30 (17–46), *p* = 0.0001], and between performing chest compression vs. no chest compression [55 (49–64) vs. 33 (18–47), *p* = 0.001].

**Conclusion:** Perceived workload of neonatal healthcare providers increases during higher acuity deliveries. Healthcare providers' workload during neonatal resuscitation can be measured using NASATLX and was inversely associated with 5-min Apgar score. Future studies assessing healthcare providers' perceived workload during neonatal resuscitation in different settings are warranted.

## Introduction

Around 10% of newborn infants require resuscitation at birth (1). Ideally, this neonatal resuscitation is provided by a team of skilled healthcare providers (HCP). This team must rapidly process various dynamic pieces of information to determine the status of the newborn and perform appropriate interventions. Human errors and deviations from protocol are common with error rates during neonatal resuscitation being between 15 and 55% (2–4). Furthermore, non-technical problems such as poor communication or breakdown of teamwork cause approximately two thirds of sentinel events during neonatal resuscitation (5). These errors may occur due to excess HCP workload. Workload refers to the cost to a human operator of completing a task and is not only related to the objective requirements of the task but is also affected by the circumstances surrounding task completion along with the skills, behaviors, and perception of the operator (6). If the workload of a task becomes too great, delays and errors become more likely, and human costs such as fatigue, stress, and illness may reach an unacceptable level (7–9). Excess workload in health care can compromise the quality and safety of patient care (7, 10).

Workload cannot be measured directly, but it can be quantified through physiological measures, secondary task performance, and subjective measurements (11). The National Aeronautics and Space Administration's Task Load Index (NASA TLX) is a frequently used subjective measure of workload (6, 12). The NASA TLX scale has been successfully used in air traffic control, aircraft, command and control centers, space operation, automobiles, and medicine (6, 12, 13).

Given the dynamic, fast-paced, and high-stakes nature of neonatal resuscitation, workload may play an important role in how HCPs perform individually and as a team. Understanding the workload experienced by HCPs during neonatal resuscitation might reveal opportunities to improve performance, decrease errors, and therefore improve patient safety. We aimed to investigate the use of NASA-TLX to evaluate HCPs' perceived workload and to determine the relationship between roles, complexity of care, and size of the healthcare team and perceived workload during neonatal resuscitation in the delivery room.

## Methods

### Study Setting

This survey was conducted between May and August 2019 at the Neonatal Intensive Care Unit (NICU), at Royal Alexandra Hospital, Edmonton, Canada. This is a level three NICU, with approximately 350 infants with a birth weight of <1,500 g annually and a total of 6,500 births per year. The Royal Alexandra Hospital Research Committee and the Health Ethics Research Board, University of Alberta (Pro00090092) approved this study. Participants were recruited through Health Ethics Research Board approved study posters, which were displayed throughout the NICU and the delivery rooms for the duration of the study period. Participation was voluntary, and consent was implied by survey submission.

### Study Participants

All neonatal HCPs (including registered nurses, transport nurses, neonatal nurse practitioners, respiratory therapists, pediatric residents, neonatal fellows, neonatologists, or medical students on their elective) who attend high-risk deliveries at Royal Alexandra Hospital were eligible to complete a survey. Every HCP was asked to complete one survey each time they attended a delivery regardless of their role, profession, level of training, or which interventions were performed at the delivery.

### Resuscitation-Stabilization-Triage Team

The Resuscitation-Stabilization-Triage (RST) team at the Royal Alexandra Hospital usually comprises two to four neonatal healthcare providers with varying levels of neonatal resuscitation skills and defined roles. A two-person RST team (registered neonatal nurse and neonatal transport nurse) attends deliveries of 29–33 weeks' gestation; for infants <29 weeks the RST team comprises a registered neonatal nurse, a neonatal respiratory therapist, and a neonatal nurse practitioner or neonatal fellow. If extended resuscitation is expected, a neonatal consultant will additionally attend the delivery.

### Survey

This was an anonymous pencil and paper survey, which took approximately 10 min to complete. Participants were asked to complete a survey immediately after delivery by taking a survey from a marked folder within the NICU. Once completed, surveys were placed into a secure letterbox and collected by the research team.

We used the NASA TLX survey, which is a subjective *post hoc* measure of workload (6). NASA TLX independently assesses six dimensions: (1) *mental demand*, which refers to how much mental or perceptual activity was required to complete the task, (2) *physical demand*, referring to the amount of physical activity required to complete the task, (3) *temporal demand*, referring to how much time pressure was experienced when completing the task, (4) *performance*, referring to the respondent's perception of how successful they were in completing their role in the task, (5) *effort*, which refers to the level of mental and physical exertion required to accomplish the level of performance achieved during the task, and (6) *frustration*, which refers to how insecure, discouraged, irritated, stressed, or annoyed respondent was during the task. The domains “mental,” “physical,” and “temporal demand” relate to the demands imposed on the participant, whereas performance, effort, and frustration focus on the interaction of the subject with the task. Each dimension is rated on a 20-step scale from “very low” to “very high,” except performance which is rated from “perfect” to “failure.” No numerical values were present on the survey scales. We translated handwritten markings to values (0–20) corresponding to the nearest step mark on the survey. To gain a better understanding of each HCP's perception of these dimensions, we also collected descriptions of factors that either contributed positively or negatively to their experience of workload. Also, we collected demographics including HCPs' related (i.e., profession, years of experience), procedural (i.e., interventions performed during resuscitation, notification time before delivery), and patient data (i.e., birth weight, Apgar score) for each delivery they attended. We aimed to collect a convenient sample of 200 surveys.

### Data Analysis

We calculated the median (interquartile range, IQR) score of each of the six workload dimensions considered in the NASA TLX. Each dimension score can range from 0–20 with 0 being the lowest and 20 the highest for mental demand, physical demand, temporal demand, effort, and frustration, and for performance 0 equated to perfect and 20 to failure. Overall, workload is represented as Raw-TLX, which was calculated for each survey response as the mean of all dimension scores multiplied by 5 to transform this score to a value out of 100. The Raw-TLX scores were compared using analysis of variance (ANOVA) with repeated measures and Bonferroni post-test. The Raw-TLX score was compared by HCP role and by the 5-min Apgar scores. The 5-min Apgar score was divided into three groups according to *Neonatal Encephalopathy and Neurologic Outcomes, Second Edition* (7–10—high Apgar score group; 4–6—medium Apgar score group; 0–3—low Apgar score group) (14). A multivariant analysis comparing the Raw LTX with the number of team members adjusted for birth weight, gestational age, 5-min Apgar score, and intervention was performed. The data are presented as mean (standard deviation, SD) for normally distributed continuous variables and median (interquartile range, IQR) when the distribution was skewed. *P*-values are two-sided, and *p* < 0.05 was considered significant. Statistical analyses were performed with Stata (Intercooled 12, Statacorp, City, Texas).

## Results

During the study period, approximately 880 neonatal resuscitation events occurred. In total, 204 surveys were completed: 105 by registered nurses (52%), 40 by transport nurses (20%), 14 by neonatal nurse practitioners (7%), 22 by respiratory therapists (10%), four by residents (R1–4) (2%), nine by neonatal fellows (4%), six by neonatologists (3%), and four by students (2%). HCP completed one survey for 179 deliveries, two surveys for 20 deliveries, and three surveys for five deliveries. HCPs had a median (IQR) of 14 (7–18) years of experience working in the NICU. Of the surveys, 168/204 were from female HCPs, 23 from male HCPs, two from HCPs who identified themselves as being neither female nor male, and 11 from HCP who did not identify their gender. Overall, HCPs' roles during resuscitation were team leader 33 (16%), airway manager 52 (26%), team leader + airway manager 16 (8%), nurse 67 (33%), respiratory therapist 15 (7%), recorder 6 (3%), observer 4 (2%), and students 11 (5%). Demographics of infants whose deliveries HCPs attended, time to prepare for delivery, and interventions performed during resuscitations are presented in Table 1. In 135 (66%) of deliveries parents were present, in 48 (24%) they were not, and in the remaining 21 surveys, HCPs did not provide an answer.

**Table 1:** Demographics of included infants, survey responses of time to prepare to attend delivery, and interventions performed during resuscitations.

**Demographics of included infants**	
Gestational age (weeks)^†^	35 (5)
Birth weight (grams)^#^	2,690 (1,830–3,440)
Apgar score 1 min	6 (4–8)
Apgar score 5 min	8 (7–9)
Female infants	85 (43%)
Cesarean section	135 (67%)
**Time to prepare to attend delivery**	**Survey responses:** ***n*** **=** **202**
Emergency code	9 (5%)
Arrived after delivery	20 (10%)
0–5min	61 (30%)
5–10 min	48 (24%)
10–20 min	28 (14%)
20+ min	36 (18%)
**Interventions performed during resuscitation**	
Stimulate	149 (73%)
Suction	130 (64%)
Continuous positive airway pressure	120 (59%)
Positive pressure ventilation	105 (52%)
Intubation	33 (16%)
Chest compression	10 (5%)
Epinephrine	4 (2%)

Data are presented as n (%), unless indicated

# *median (IQR)*,

† *mean (SD)*.

### Dimension Score

**Figure 1:**
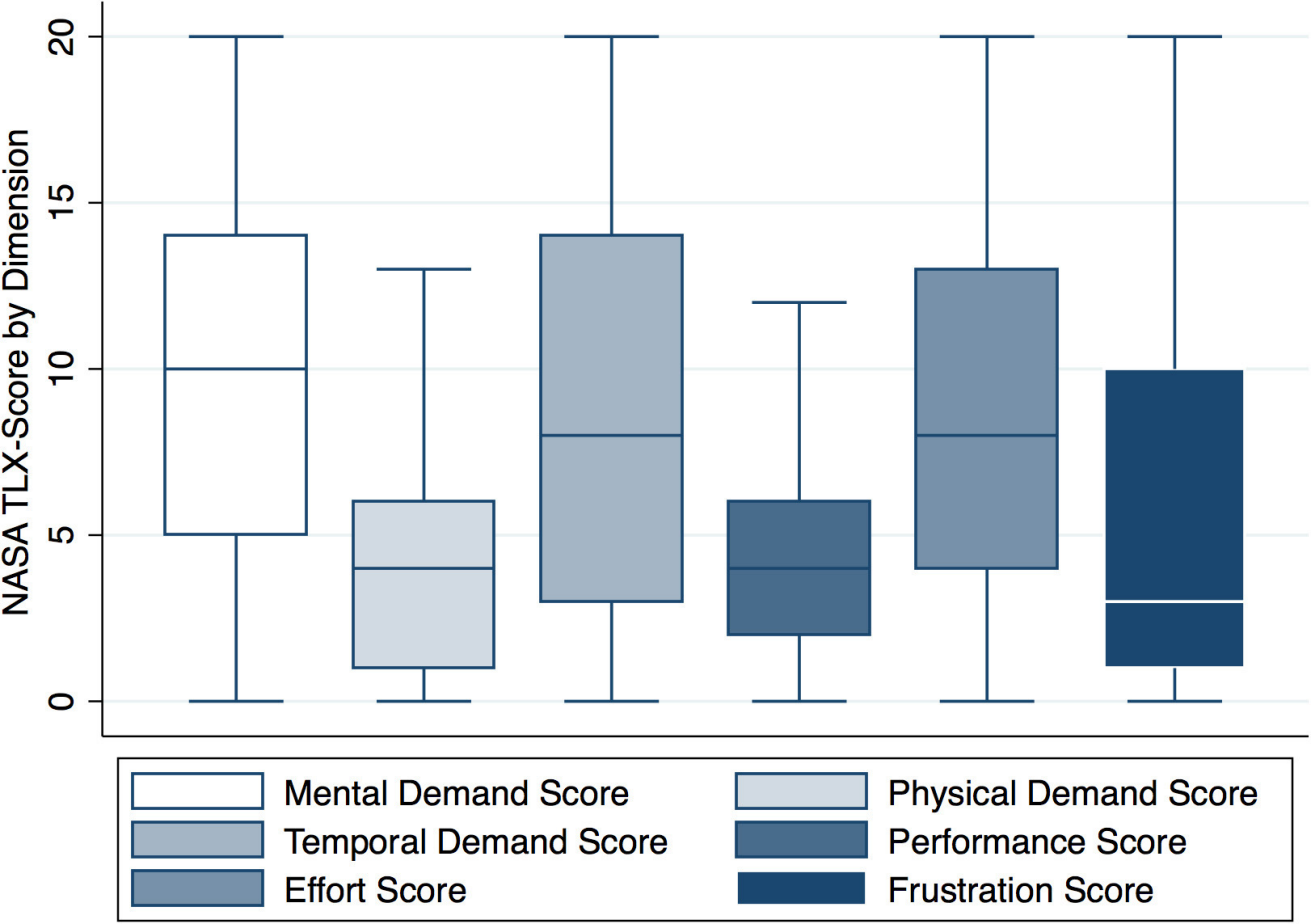
NASA TLX score by dimension. NASA TLX scores by dimension (*y* axis). Box plots represent median values (solid bar), IQR (margins of box), and whiskers as 95% confidence interval.

The median (IQR) dimension score (out of 20) of mental demand was 10 (5–14), for physical demand 4 (1–6), for temporal demand 8 (3–14), for performance 4 (2–6), for effort 8 (4–13), and for frustration 4 (1–10) (Figure 1). The dimension scores, the sum of the dimension score, and Raw-TLX divided by HCP's role during resuscitation are presented in Table 2. Description of workload dimensions are presented in Table 3.

**Table 2:** Dimension score by role, sum of dimension score, and raw TLX score.

	**Mental demand score**	**Physical demand score**	**Temporal demand score**	**Performance**	**Effort**	**Frustration**	**Sum of dimension scores**	**Raw TLX score**
Team leader (*n* = 49)	8 (4–15)	3 (1–5)	5 (2–14)	3 (2–5)	7 (3–12)	2 (1–9)	32 (15–55)	27 (13–46)
Airway manager (*n* = 66)	10 (6–15)	4 (2–6)	7 (3–13)	4 (2–5)	9 (4–14)	3 (1–8)	40 (25–56)	33 (21–47)
TL + AM (*n* = 16)	10 (6–15)	3 (2–6)	7 (2–14)	4 (3–5)	10 (4–13)	3 (1–10)	38 (21–55)	31 (17–46)
Registered nurse (*n* = 69)	10 (3–14)	4 (1–7)	8 (3–14)	4 (2–6)	8 (4–14)	3 (1–11)	41 (22–58)	34 (18–48)
Respiratory therapist (*n* = 15)	12 (8–14)	4 (3–5)	15 (11–18)	5 (4–12)	9 (7–14)	8 (4–14)	59 (46–74)	49 (38–62)
Recorder (*n* = 6)	13 (9–16)	4 (5–6)	13 (8–14)	5 (4–7)	7 (5–11)	8 (1–12)	52 (42–59)	43 (35–49)
Observer (*n* = 4)	5 (2–16)	2 (1–3)	9 (5–14)	3 (2–4)	4 (3–5)	3 (2–3)	20 (15–36)	16 (12–30)
Student (*n* = 12)	10 (5–13)	5 (2–7)	10 (4–11)	4 (3–6)	10 (6–14)	6 (2–10)	42 (30–56)	35 (25–46)

*Data are presented as median (IQR); TL + AM, team leader + airway manager*.

**Table 3:** Description of workload dimensions considered in NASA TLX adapted from NASA TLX paper and pencil version instruction manual (14).

**Title of dimension**	**Endpoints**	**Description provided**	**Example of contributor**
Mental demand	Low-high	How much mental and perceptual activity was required	“Active resuscitation—looking/assessing many different things and listening to instructions from team lead” “First delivery where I recorded so lack of knowledge was main contributor”
Physical demand	Low-high	How physically demanding was the task? Was the task easy or demanding, slow or brisk, slack or strenuous, restful or laborious?	“Room too small no place to put supplies” “Running to set Viasys up in a timely fashion”
Temporal demand	Low-high	How much time pressure did you feel due to the rate or pace at which the task or task element occurred? Was the pace slow and leisurely or rapid and frantic?	“Heart rate was low and not responding” “Calm organized team, senior”
Performance	Good-poor	How successful do you think you were in accomplishing the goal of the task? How satisfied were you with your performance in accomplishing these goals?	“Good NRP (neonatal resuscitation protocol) and teamwork” “Experience allowing me to anticipate what would need to be done”
Effort	Low-high	How hard did you have to work mentally and physically to accomplish your level of performance?	“It was late I was tired” “New to transport roll still building confidence in role especially when performing resuscitation without 2nd NICU team member”
Frustration	Low-high	How insecure, discouraged, irritated, stressed, and annoyed vs. secure and complacent did you feel during the task	“Team made it easy despite difficult issues” “More frustrated with lack of communication from labor and delivery and unpreparedness”

### Raw-TLX Score

**Figure 2:**
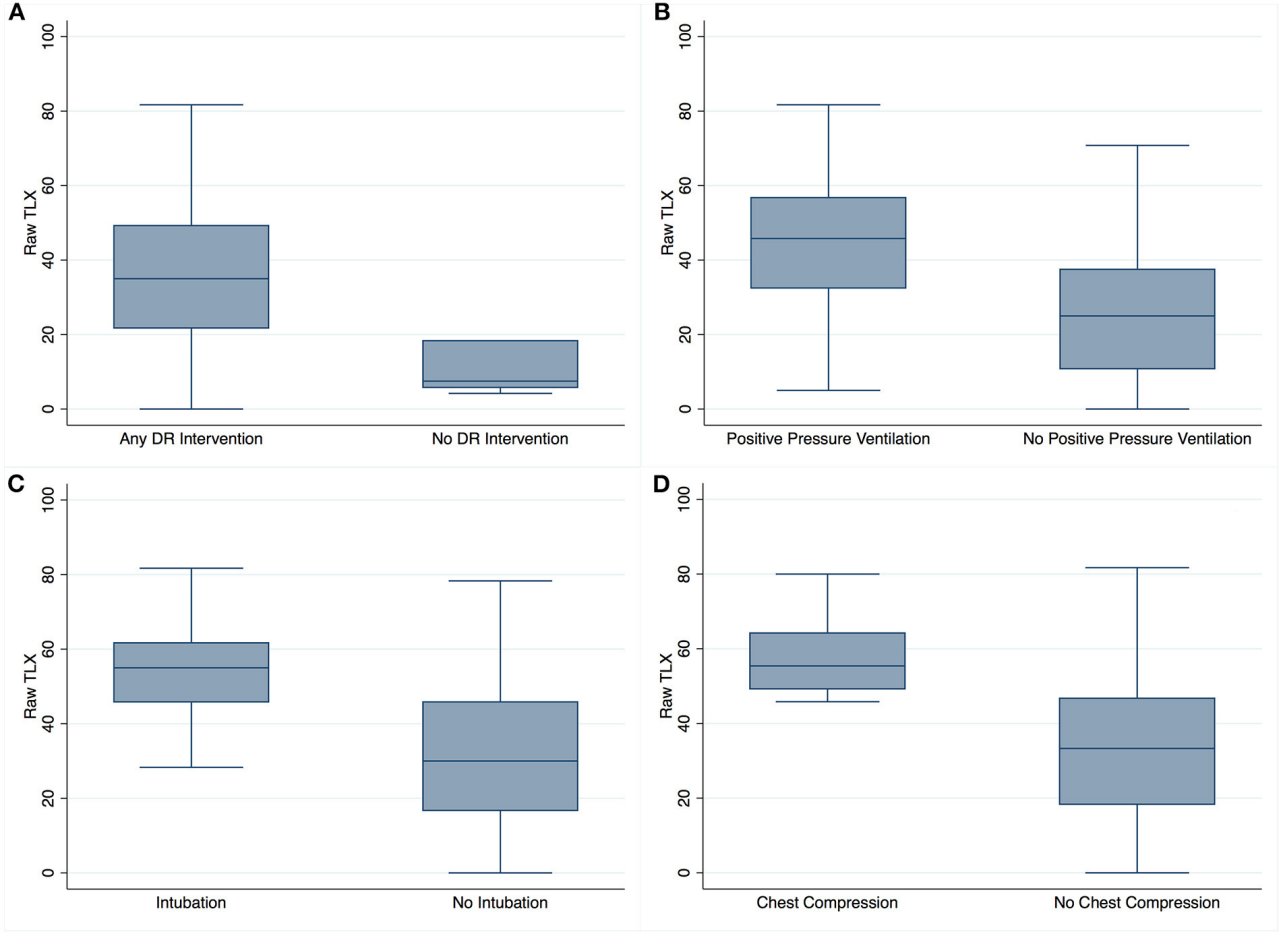
Raw-TLX scores based on intervention performed. Raw-TLX scores (*y* axis) of healthcare providers attending deliveries in which the baby either required **(A)** any delivery room (DR) intervention vs. no delivery room (DR) intervention, **(B)** positive pressure ventilation (*n* = 105) vs. no positive pressure ventilation (*n* = 99), **(C)** intubation (*n* = 33) vs. no intubation (*n* = 171), and **(D)** chest compression (*n* = 10) vs. no chest compression (*n* = 194). Box plots represent median values (solid bar), IQR (margins of box), and whiskers as 95% confidence interval.

The overall median (IQR) Raw-TLX (overall workload score) was 34 (18–49). The Raw-TLX was higher when HCPs performed any interventions compared to no interventions with 35 (22–49) vs. 8 (6–18), *p* = 0.0011 (Figure 2A). Furthermore, the Raw-TLX was higher during positive pressure ventilation vs. no positive pressure ventilation with 46 (33–57) vs. 25 (11–38), *p* = 0.0001 (Figure 2B), similar with intubation vs. no intubation 55 (46–62) vs. 30 (17–46), *p* = 0.001 (Figure 2C), and chest compression vs. no chest compression 55 (49–64) vs. 33 (18–47), *p* = 0.001 (Figure 2D).

The Raw-TLX in the low 5-min Apgar score group was 54 (48–61), compared to the medium or high 5-min Apgar score groups with 47 (36–58) and 28 (14–28), respectively. One-way ANOVA revealed significantly higher Raw-TLX in the low and medium 5-min Apgar score groups compared to the high 5-min Apgar score groups (*p* = 0.001).

**Figure 3:**
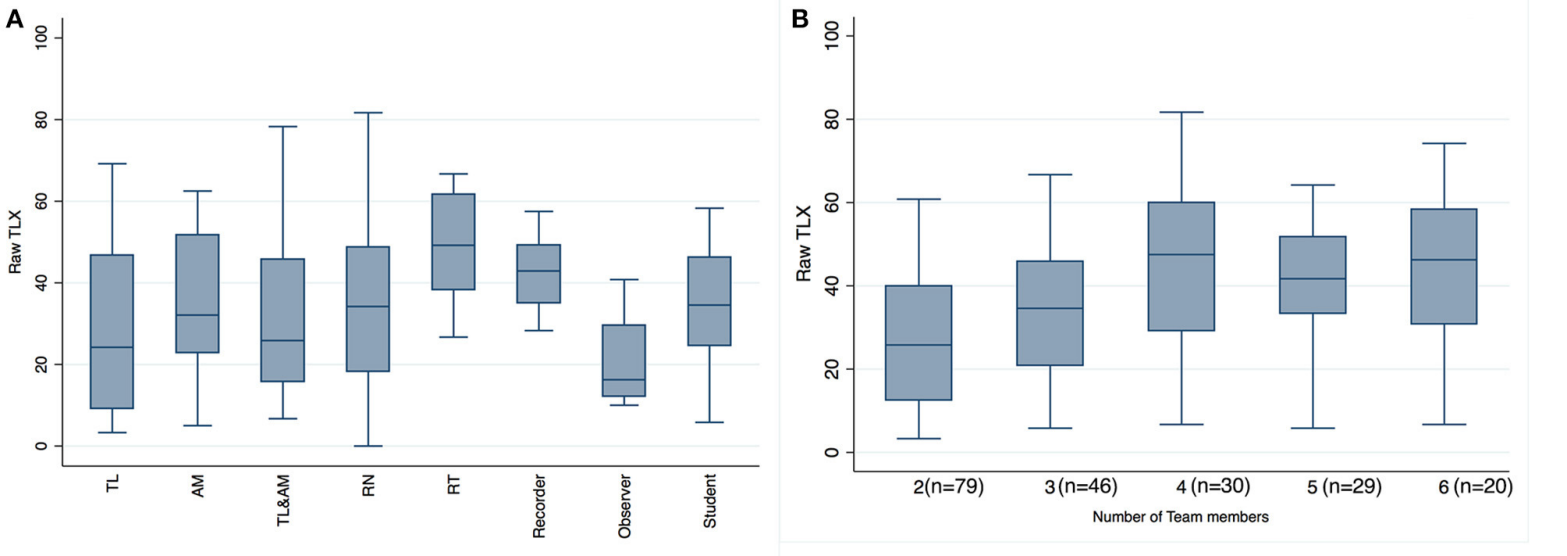
Raw-TLX scores by provider role in resuscitation and by number of team members. Raw-TLX scores (*y* axis) of healthcare providers **(A)** by role during resuscitation **(B)** by number of team members attending the delivery. TL, team lead; AM, airway manager; TL&AM, team lead and airway manager; RN, registered nurse; RT, respiratory therapist. Box plots represent median values (solid bar), IQR (margins of box), and whiskers as 95% confidence interval.

Overall, the Raw-TLX was similar regardless of HCP roles during resuscitation (Figure 3A). Multivariant analysis revealed an increase in Raw-TLX until the healthcare team reached a size of four team members (*p* = 0.0001) (Figure 3B).

## Discussion

To our knowledge, this is the first study using the NASA TLX to examine workload of HCPs during neonatal resuscitation in the delivery room. Overall workload varied largely within roles but was similar between roles (Figure 1). There was a significantly increased overall workload when HCPs cared for infants with a lower 5-min Apgar score or with escalating delivery room interventions (Figure 2). With increasing team size, there was an increase in workload for up to a total of four team members (Figure 3).

NASA TLX is a subjective measure of workload that has been validated in medicine (i.e., intensive care unit nurses and pediatric trauma resuscitations), aeronautics, psychology, and driving (12, 15, 16). A meta-analysis reporting the NASA TLX scores for tasks from a variety of disciplines including medicine, sports, aeronautics, and psychology described a mean overall workload score of 45 (15) in all tasks (17). In the same meta-analysis, Grier et al. reported the median reported NASA global score for medical tasks was 50.6 (39–61). NASA global scores in this meta-analysis were calculated in two different ways (Raw-TLX as calculated in this study and a weighted scoring system) which have been shown to correlate closely (17). The overall Raw-TLX score in our study was 34 (18–49), which may suggest that this task is associated with a lower perceived workload than other medical tasks. These differences may be due to the demands of the task, resources available in our institution, or experience level of our participants. Additionally, the objective task demands of delivery room events included in this study varied largely from no intervention to complex and high-pressure interventions (e.g., intubation and chest compression). This degree of variability in task difficulty potentially exceeds that of other medical tasks.

The paper and pencil NASA TLX survey was simple, convenient, and rapidly completed by respondents. We have demonstrated the feasibility of NASA TLX and supported its validity in HCPs participating in neonatal delivery room care as Raw-TLX increased significantly with the complexity of care required, as expected. Raw-TLX was significantly higher in HCPs who attended the delivery of infants with low 5-min Apgar scores 54 (48–61), compared to infants with medium 38 (30–48) or high 25 (13–40) 5-min Apgar scores. Furthermore, we found a significantly higher perceived workload for HCPs who attended deliveries of infants who required any delivery room intervention compared to no delivery room intervention (Figure 2A). Similarly, positive pressure ventilation, intubation, and chest compression were independent variables, which significantly increased the perceived workload (Figures 2B–D). These findings suggest that HCPs experience higher workload with an increased level of care, which might be due to increased (i) objective task demands, (ii) perception of the need for rapid interventions, and (iii) perceived significance of potential consequences of delays or errors. Furthermore, HCPs might have had less experience participating in high-level resuscitations (high acuity, low occurrence events), which might have added to the perceived workload.

The overall workload varied largely within the assigned roles during delivery room care but was similar between roles (Figure 3A). This suggests that workload during delivery room care either was similar between roles among the healthcare team or could reflect an insufficient sample size due to a large number of different roles. Interestingly, HCPs who acted as team lead + airway manager reported similar Raw-TLX scores to HCPs who acted as either team leader or airway manager despite holding two roles simultaneously even when we controlled for Apgar score and the number of interventions applied. The similar Raw-TLX scores between the dual-role and single-role cases might reflect a decrease in role confusion and therefore decreased demands on the combined role. Previous assessments of HCP workload have demonstrated variable results in terms of workload distribution across roles. Tofil et al. reported that team leaders perceived a greater workload compared to their team during simulated sepsis scenario (18), while Geis et al. observed that medication nurses perceived greater workload than their team members during simulated pediatric resuscitations (19). Understanding the workload distribution between team members and its effect on each team member's performance will allow us to design targeted interventions to improve equitable role assignment and workload management.

### Limitations

By collecting surveys in an anonymous and self-administered manner, we were able to collect surveys 24 h a day, which has been cited as a challenge in previous applications of NASA TLX in critical care settings (16). However, this method of survey collection did not allow us to record the survey response rate. Additionally, recall bias might have resulted in inaccuracies in reporting of the performed interventions and their perceived workload. Although HCPs should have completed surveys immediately after returning from each delivery, due to the busy nature of the NICU, some of the surveys might have been completed several hours after the delivery room event. NASA TLX guidelines suggest that surveys be completed within 25 min of task completion; we do not know the exact time frame HCPs completed each survey. Furthermore, some HCPs might have only completed surveys for deliveries that they perceived to require a high workload, which may have limited the representativeness of our results. Finally, our analysis was limited by our inability to pair repeated survey responses by a single HCP due to the survey's anonymity. Therefore, each survey response was treated as an independent variable in our analysis despite some HCPs completing several responses.

Caution should be taken to generalize this result into other delivery room settings, as workload is also dependent on available resources, social settings (i.e., safety culture), and participants' level of training. In addition, there was a very low number of physician response, which is due to the setup of our RST team with primary attendance of transport nurses, respiratory therapists, and neonatal nurse practitioners. The participants in this study work in a high-risk delivery room setting and perform neonatal resuscitation tasks daily, which might have resulted in a lower level of workload compared to a novice group of HCPs or HCPs participating in resuscitations less frequently.

## Conclusion

Workload can be assessed using the NASA TLX during neonatal delivery room care. Workload was increased during deliveries with higher acuity, with escalating delivery room interventions, and increased number of team members. Future research should assess the HCPs' workload to optimize patient safety, team qualifications, and interventions that reduce excess workload.

## Data Availability Statement

The original contributions generated for this study are included in the article/supplementary materials, further inquiries can be directed to the corresponding author/s.

## Ethics Statement

The Royal Alexandra Hospital Research Committee and the Health Ethics Research Board, University of Alberta (Pro00090092) approved this study. The patients/participants provided their written informed consent to participate in this study.

## Author Contributions

EZ, BL, and GS: conception, design, collection, assembly of data, analysis, interpretation of the data, drafting of the article, and critical revision of the article for important intellectual content. All authors contributed to the article and approved the submitted version.

## Conflict of Interest

The authors declare that the research was conducted in the absence of any commercial or financial relationships that could be construed as a potential conflict of interest.
